# Circulating immune complexes and mutations of HBsAg are associated with the undetectable HBsAg in anti-HBs and HBeAg positive occult hepatitis B virus infection

**DOI:** 10.3389/fmicb.2022.1063616

**Published:** 2022-11-29

**Authors:** Ying Yan, Huizhen Sun, Le Chang, Huimin Ji, Xinyi Jiang, Shi Song, Yingzi Xiao, Kaihao Feng, Abudulimutailipu Nuermaimaiti, Zhuoqun Lu, Lunan Wang

**Affiliations:** ^1^National Center for Clinical Laboratories, Institute of Geriatric Medicine, Chinese Academy of Medical Sciences, Beijing Hospital/National Center of Gerontology, Beijing, China; ^2^Beijing Engineering Research Center of Laboratory Medicine, Beijing, China; ^3^Graduate School, Peking Union Medical College, Chinese Academy of Medical Sciences, Beijing, China

**Keywords:** occult HBV infection, circulating immune complexes, immune escape mutations, Lumipulse HBsAg-HQ assay, HBeAg

## Abstract

**Introduction:**

Occult hepatitis B virus infection (OBI) is an HBsAg negative state in HBV infection with usually inactive HBV replication. However, there were a minority of individuals with positive HBeAg and anti-HBs among OBI blood donors and few studies have focused on this unusual serological pattern.

**Methods:**

2022 plasma of blood donors that preliminary screened reactive for HBV DNA and non-reactive for HBsAg were collected from 16 provinces in China from 2015 to 2018. HBV DNA and HBsAg in these samples were retested using the Cobas TaqScreen MPX test and ARCHITECT HBsAg Quantitative II assay. Lumipulse HBsAg-HQ assay and polyethylene glycol (PEG)-double precipitation following HCl and trypsin digestion were performed to detect HBsAg from HBsAg-anti-HBs circulating immune complexes (CICs).

**Results:**

1487 of 2022 samples were positive for Cobas HBV DNA test and non-reactive for ARCHITECT HBsAg assay, while 404 of them were positive using Lumipulse HBsAg-HQ assay. 10 HBsAg-/anti-HBs+/HBeAg+ OBI blood donor samples were further dissociated and HBsAg-CICs were detected in 7 samples. Sequencing analysis showed that D44N, N98T, G73S, Del 56-116, and I161T occurred in the pre-S region, and immune escape mutations such as P127T, F134L, G145R, V168A, and I126T/S in the S region were found.

**Discussion:**

In conclusion, there were a minority of HBsAg-/anti-HBs+/HBeAg+ individuals in OBI blood donors. The undetectable HBsAg in these individuals was mainly due to HBsAg-CICs. Immune escape-associated mutations also happened under the host’s selective pressure. HBsAg dissociation methods or Lumipulse HBsAg-HQ assay is recommended to distinguish these individuals.

## Introduction

Hepatitis B, a severe liver infection caused by the hepatitis B virus (HBV), continues to be a global public health issue that requires an urgent response. HBV infection can be chronic, and significantly increases the risk of developing liver cirrhosis (LC), hepatocellular carcinoma (HCC), and even death (Papatheodoridis et al., 2015; Xie, 2017; Shih et al., 2018). The detection of HBV serological markers is an effective method to diagnose HBV infection and establish the stage of infection. The typical serological pattern of chronic HBV infection is the presence of circulating hepatitis B surface antigen (HBsAg) (Coffin et al., 2019). Different from the overt infection, occult HBV infection (OBI) is a special state in HBV infection, which is defined as the presence of replication-competent HBV DNA in the liver and/or HBV DNA in the blood of individuals with negative HBsAg results using currently available assays (Mak et al., 2020). Thus, the sensitivity of the test utilized affects how well OBI is recognized. Due to the invasion of liver biopsy, circulating HBV DNA without detectable HBsAg, combined with follow-up is commonly used to identify OBI in clinical practice.

Based on the serological results, OBI may be categorized as seropositive OBI and seronegative OBI, the former represents hepatitis B core antibody (anti-HBc) and/or HBsAg antibody (anti-HBs) positive, the latter denotes the absence of both anti-HBc and anti-HBs (Raimondo et al., 2019). HBV replication activity is typically dormant in the majority of OBI individuals, with the exception of certain cases in which the undetected HBsAg is brought on by S-eacape mutations. However, we identified a small subset of OBI blood donors that were positive for HBeAg, a marker of active HBV replication. HBsAg negative appears to contradict virus’s active replication status represented by HBeAg positive, and may result in clinical neglect of these individuals, affecting their diagnosis, treatment, and prognosis.

In the present study, we identified ten OBI blood donors with HBsAg-/anti-HBs+/HBeAg+ serological pattern who had never received anti-viral treatment. Through detecting samples using Lumipulse HBsAg-HQ assay which could dissociate and detect HBsAg from circulating immune complexes (CICs) in the plasma samples, we found that the undetectable HBsAg was mainly caused by the formation of HBsAg-CICs. By sequencing the HBsAg encoding area, HBsAg variants under the influence of the host immune system were also discovered, which may also be associated with the undetected HBsAg. This study highlights an unusual infection status in OBI, which is helpful in deeply understanding the complexity and diversity of virus-host interactions in HBV infection. Meanwhile, more clinical attention should be paid to these individuals to avoid disease progression.

## Materials and methods

### Objects of study and testing

From 2015 to 2018, 2022 HBV DNA reactive and HBsAg non-reactive plasma samples were collected from 39 blood centers in 16 provinces of China. These samples were all derived from blood donors, a population that was unaware of the infection and never received antiviral treatment. The presence of HBV DNA was confirmed using the Roche screening reagent of blood nucleic acid (Cobas TaqScreen MPX test, v2.0; Roche Molecular Systems, Inc., NJ, USA). Serological markers of HBV infection were retested by the Architect-i2000 chemiluminescence immune analyzer and the corresponding reagents (ARCHITECT HBsAg Quantitative II, anti-HBs, HBeAg, anti-HBe, and anti-HBc) (Abbott Laboratories, Abbott Park, IL, USA). The ARCHITECT HBsAg Qualitative II assay is a one-step immunoassay with a cutoff value of ≥1 S/CO.

In addition, HBsAg was also measured on the two-step sandwich Lumipulse HBsAg-HQ assay (Cat. no.291153, Fujirebio, Inc. Tokyo, Japan) with a fully automated chemiluminescent enzyme immunoassay system (Lumipulse G1200; Fujirebio, Inc., Tokyo, Japan). Briefly, samples were pretreated with a solution that includes surfactant to disrupt HBV particles, to dissociate HBsAg from HBsAg-anti-HBs complexes, and to denature epitopes to a linear form. Then, HBsAg was detected using two monoclonal antibodies against external structural regions (“a” determinant) and the internal epitopes (28–80 amino acids, aa) as a capture reagent, with two monoclonal antibodies coupled to alkaline phosphatase as the detector (Shinkai et al., 2013; Deguchi et al., 2018). HBV S protein is a transmembrane protein, and the internal epitopes are located within the viral particles. Even in the HBsAg-CICs, internal epitopes do not expose and conjugate to anti-HBs in the human body. After denaturing epitopes to a linear form, the monoclonal antibodies, especially the one against the internal epitopes could combine HBsAg as a capture. With two monoclonal antibodies coupled to alkaline phosphatase as the detector, HBsAg in HBsAg-CICs could be detected. The analytical sensitivity of this assay is 0.005 IU/ml.

Confirmation of positive results from Lumipulse HBsAg-HQ assay was performed by neutralization assay (also called inhibition assay) following the manufacturer’s instructions. Briefly, 30 μL of HBsAg-HQ Inhibitory antibody solution was added to specimen (A). As a control, 30 μL of HBsAg-HQ Inhibitory control solution was added to specimen (B). Each mixture was left at 20–30°C for 10–60 min and the HBsAg-HQ assay was then carried out. When the value of (B-A)/B ≥ 50%, the test was determined to be positive.

Cboas AmpliPrep/COBAS TaqMan HBV Test, v2.0 (Roche Molecular System, Inc., NJ, USA) was used to quantify HBV DNA load.

### Ethics approval

This study is approved by the Bioethics Committees of Beijing Hospital. The reference number is 2019BJYYEC-228-02.

### Detection of circulating immune complexes

#### Preparation of circulating immune complex dissociation reagent

The 0.15 M borate buffer containing 0.132 M NaCl was added with 8 and 7% polyethylene glycol (PEG) 6000 to produce CIC separating solution A and CIC separating solution B, respectively (Dai et al., 2018; Wang et al., 2019). The parameters for the quality control were a pH of 8.40–8.80 with the osmolarity at 500–530 osm.

#### Circulating immune complex positive control

Positive control was prepared by incubating the standard material for HBsAg and anti-HBs (Abbott Laboratories, Abbott Park, IL, USA). Briefly, the mixture of HBsAg (50 IU/ml) and anti-HBs (1000 mIU/ml) was incubated at 37°C for 2 h and then at 4°C overnight.

#### Double-precipitation separation

200 μL of CIC separating solution A was mixed with 200 μL of plasma sample or positive control in a 1.5 ml-tube, followed by incubation at 37°C for 30 min and then at 4°C for at least 6 h. The mixture was centrifuged at 4000 *g* for 20 min, and the supernatant was removed. 200 μL of 0.9% NaCl was added to the above CIC sediment and vortexed until the granules were dissolved. 200 μL of CIC separating solution B was added, followed by incubation at 4°C for at least 6 h. The mixture was centrifuged at 4000 *g* for 20 min and the supernatant was removed.

#### HBsAg dissociation from circulating immune complexes

The CIC sediment from double-precipitation separation was dissolved in 20 μL of 0.9% NaCl and equally divided into two tubes, which were labeled as the corresponding test tube and blank control tube. The test (T) tube was added 50 μL of 0.6M HCl and 50 μL of trypsin (10 g/L), followed by incubation at 37°C for 30 min, and then added 100 μL of 0.08M Tris to end the reaction. The control (C) tube was straightforwardly added 50 μL of 0.6M HCl and 50 μL of trypsin (10 g/L) and 100 μL of 0.08M Tris without incubation at 37°C. The HBsAg in two tubes was quantitated using the Abbott i2000 analyzer and ARCHITECT Alinity i HBsAg Reagent (Abbott Laboratories, Abbott Park, IL, USA). The analytical sensitivity of this assay is 0.05 IU/ml. The HBsAg titers of the test tube and control tube were referred to as HBsAg-T and HBsAg-C, respectively. Presence of CICs was calculated as the titer of HBsAg-T tube minus HBsAg-C tube, and expressed as HBsAg T-C. The criteria for the presence of CICs were HBsAg-T > 0.05 IU/ml and HBsAg T-C > 0 IU/ml.

### Sequencing of hepatitis B virus pre-S/S region

#### Ultracentrifugation

Ultracentrifugation was conducted to enrich the HBV in samples with relatively low viral loads. Briefly, a 7–8 ml plasma sample was added into a centrifuge tube and was centrifuged at 50,000 rpm/min for 4 h by the ultracentrifuge (Thermo Scientific Inc., Sorvall™ WX+, Germany). The supernatant was discarded after centrifugation, and 700 μL of proteinase K-PBS solution (proteinase K purchased from Takara Bio Inc., Kusatsu, Japan) was added to resuspend the precipitation, followed by incubating at 50°C for 12–15 h, and then DNA was extracted from the lysed sample.

#### DNA extraction from plasma samples, PCR amplification, cloning, and sequencing

DNA was extracted using the Nucleic Acid Extraction Kit (PerkinElmer^®^, Waltham, MA, USA) and subjected to nested PCR amplification of HBV pre-S/S region. The primers were listed in Supplementary Table 1. Briefly, PCR was carried out in 25 μL of a reaction mixture including 10 μL DNA template, 0.625U Amplitaq DNA polymerase (Applied Biosystems, Carlsbad, CA, USA), 1 × PCR buffer, 0.1 mM dNTP mix and 0.2 μM primer. Two microliters aliquot of the first-round PCR products was used as the template of the second-round PCR. Amplification was in the following steps: initial denaturation at 95°C for 2 min, 35 cycles (first round) or 40 cycle (second round) of 95°C for 15 s, 53.5°C (pre-S) or 55°C (S) for 30 s, and 72°C for 1 min (pre-S) or 1.5 min (S), then incubation at 72°C for 5 min.

Following the PCR amplification and subsequent electrophoresis, the PCR products (pre-S and S amplicons) were recovered and purified from agarose gel using a gel extraction kit (TIANGEN, Beijing, China). The purified PCR product of each sample was cloned into the pEASY-T1 vector (TransGen Biotech, Beijing, China). Sequencing was performed with the cloned DNA using the vector’s universal primers (Supplementary Table 1).

#### Sequence analysis

The HBV pre-S/S sequences obtained were compared with the reference sequences in the Genbank for HBV genotyping. Phylogenetic analysis was performed using the neighbor-joining method (MEGA software, version 7). The consensus sequence of genotype B or genotype C sequences was regarded as the reference sequence for analyzing genetic variants (Genotype B: Genbank accession number FJ386582–386688; genotype C: Genbank accession number FJ562218-562340).

### Statistical analysis

Statistical analysis was performed by the χ^2^ test or Fisher’s exact test for categorical variables. Mann–Whitney test was performed to compare the differences of quantitative results between two groups. Statistical processing was carried out by IBM SPSS Statistics Version 23.0 (SPSS Inc, Chicago, IL, USA), The differences were considered statistically significant when the value of *P* < 0.05.

## Results

### Characteristics of HBsAg-/anti-HBs+/HBeAg+ OBI samples in blood donors

**FIGURE 1:**
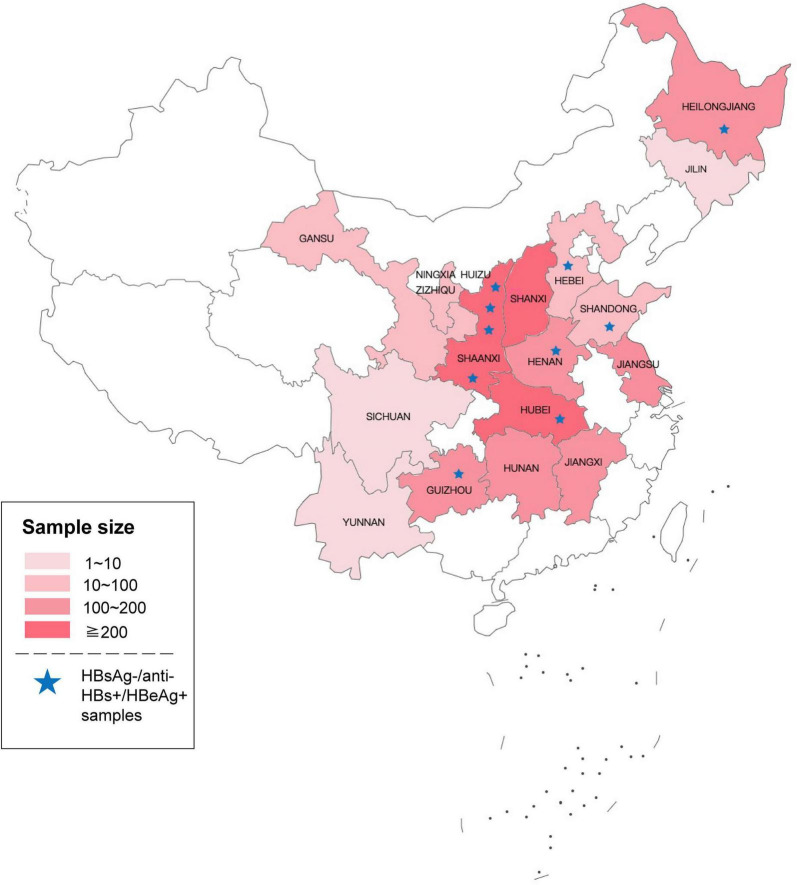
Geographical distribution of the 2022 samples that preliminary screened reactive for HBV DNA and non-reactive for HBsAg. Asterisks represent HBsAg-/anti-HBs+/HBeAg+ samples tested by ARCHITECT reagents (The original map is from the Map World of National Platform for Common Geospatial Information Services).

From 2015 to 2018, 2022 HBV DNA reactive and HBsAg non-reactive plasma of blood donors were collected from 39 blood centers and central blood stations in 16 provinces in China. As seen in Figure 1, these samples were mainly distributed in the central part and northeast of China. Among them, 1,509 samples were retested positive for HBV DNA using the Cobas^®^ TaqScreen MPX test. Of those HBV DNA-positive samples, 1,487 were non-reactive for ARCHITECT HBsAg Quantitative II assay. To further confirm the presence of HBsAg, all samples were tested by Lumipulse HBsAg-HQ assay, which could dissociate HBsAg from HBsAg-anti-HBs complexes and target both the external structural regions (“a” determinant) and the internal epitopes (28–80 aa) of HBsAg (Shinkai et al., 2013). Four hundred four HBsAg non-reactive samples were positive in the HBsAg-HQ assay. Among them, 172 samples were randomly selected and further tested by HBsAg confirmatory neutralization assay, and 170 of them were confirmed positive. In addition, HBeAg and anti-HBs were also tested. Interestingly, we found 10 samples with detectable HBeAg and anti-HBs while HBsAg was not detected using ARCHITECT HBsAg Quantitative II assay. Of the 10 HBsAg-/anti-HBs+/HBeAg+ samples, seven tested positive by Lumipulse HBsAg-HQ assay. This result prompted that the undetected HBsAg in these samples may be due to the presence of HBsAg-anti-HBs complexes.

**FIGURE 2:**
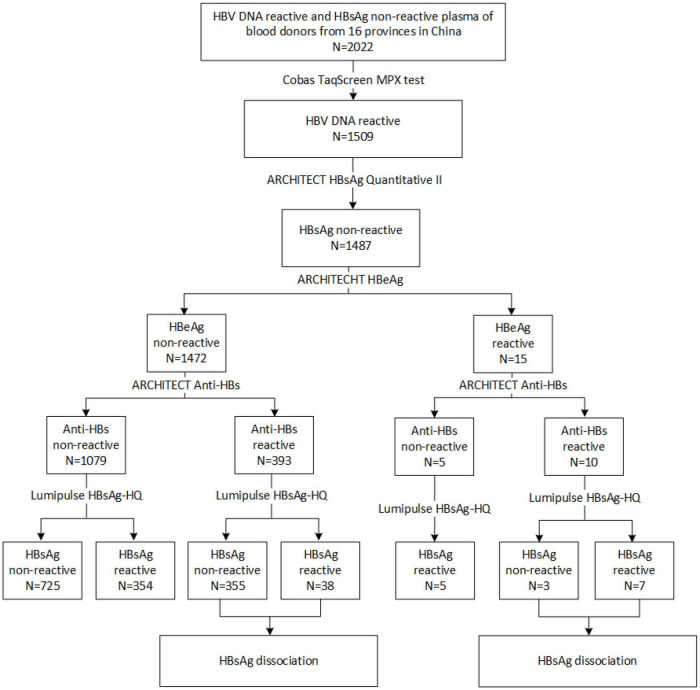
Flow chart for identifying HBsAg-/anti-HBs+/HBeAg+ samples.

To explore the reasons for this serological pattern, 6 of 10 with enough volume were dissociated and sequenced (Figure 2). Four additional samples without Lumipulse HBsAg-HQ results, but with an HBsAg-/anti-HBs+/HBeAg+ pattern tested by ARCHITECT reagents, were also included in the further study. Characteristics of the ten samples were summarized in Table 1. The HBV viral load of sample No.1 was 8,530 IU/ml. Six samples were between 40 and 300 IU/ml, and the other three were below 20 IU/ml. Six samples were genotype C and four were genotype B. Adrq+ was the most common serotype in these samples, and different serotype strains coexisted in samples 4 and 8, which may be associated with the HBsAg variants or different strains coinfection. In addition, 26 HBsAg-/anti-HBs+/HBeAg- samples were also dissociated as control, and only one of the 26 samples tested positive for HBsAg using Lumipulse HBsAg-HQ assay. Characteristics of the 26 samples were listed in Supplementary Table 2.

**TABLE 1:** Characteristics of the 10 HBsAg-/anti-HBs+/HBeAg+ samples.

Sample code	Genotype	Serotype	HBV DNA (IU/ml)	HBsAg-HQ (IU/ml)	HBsAg (S/CO)	Anti-HBs (mIU/ml)	HBeAg (S/CO)	Anti-HBe (S/CO)	Anti-HBc (S/CO)
1	B	Adw3	8530	R (0.144)	N (0.42)	R (176.89)	R (123.33)	N (7.59)	R (8.72)
2	C	Adrq+	284	R (0.047)	N (0.21)	R (>1000)	R (12.97)	N (2.28)	R (8.38)
3	C	Adrq+	235	N (0.004)	N (0.22)	R (34.16)	R (2.28)	N (1.80)	R (3.82)
4	C	Ayw1, Ayr	219	R (0.069)	N (0.23)	R (178.72)	R (7.01)	N (1.74)	R (7.87)
5	B	Adw2	235	NA	N (0.22)	R (434.01)	R (44.92)	N (3.89)	R (6.74)
6	C	Adrq+	<20	R (0.061)	N (0.26)	R (34.37)	R (3.80)	N (1.86)	R (7.05)
7	C	Adrq+	<20	R (0.194)	N (0.24)	R (107.21)	R (2.89)	N (1.53)	R (10.34)
8	B	Adw3, Ayw3	46.9	NA	N (0.21)	R (48.85)	R (8.51)	N (1.93)	R (9.65)
9	C	Adrq+	51	NA	N (0.37)	R (109.98)	R (2.88)	N (1.45)	R (7.19)
10	B	Adw2	<20	NA	N (0.87)	R (27.13)	R (16.82)	N (2.11)	R (7.59)

NA, not available.

### The presence of HBsAg-CICs in HBsAg-/anti-HBs+/HBeAg+ samples

Circulating immune complexes in HBsAg-/anti-HBs+/HBeAg+ plasma was firstly separated by the double-precipitation method using PEG6000. Then HBsAg in CICs was dissociated by treated with HCl and trypsin and quantitated by ARCHITECT HBsAg reagent. HBsAg was detected in seven of the ten samples, indicating the presence of CICs (Table 2). The viral load between the CIC positive and negative groups has no significant difference compared by the Mann–Whitney test [2.02 (1.41–2.42) log10 IU/ml vs. 2.37 (2.02, 2.37) log10 IU/ml, *p* = 0.85]. As for the 26 HBsAg-/anti-HBs+/HBeAg- OBI samples, none of them detected HBsAg-CICs (Supplementary Table 2). Fisher’s exact test showed that the percentage of samples which could dissociate HBsAg in the HBsAg-/anti-HBs+/HBeAg+ group was significantly higher than that of controls (70% vs. 0%, *P* < 0.001). These results were consistent with the results of Lumipulse HBsAg-HQ assay and demonstrated that the presence of HBsAg-CICs might account for the undetectable HBsAg in HBsAg-/anti-HBs+/HBeAg+ samples.

**TABLE 2:** HBsAg-CICs detection of the 10 HBsAg-/anti-HBs+/HBeAg+ samples.

	HBsAg dissociation^a^			Lumipulse
	
Sample code	HBsAg-T (IU/ml)	HBsAg-C (IU/ml)	HBsAg T-C (IU/ml)	HBsAg-HQ (IU/ml)
1	0.26	0.16	R (0.1)	R (0.144)
2	0.13	0.03	R (0.1)	R (0.047)
3	0.01	0.01	N (0)	N (0.004)
4	0.1	0.03	R (0.07)	R (0.069)
5	0.02	0	N (0.02)	NA
6	0.6	0.18	R (0.42)	R (0.061)
7	0.23	0.15	R (0.08)	R (0.194)
8	0.01	0.01	N (0)	NA
9	1.51	0.14	R (1.37)	NA
10	0.13	0.09	R (0.04)	NA

^a^HBsAg-T and HBsAg-C represent the HBsAg titer of the test tube and control tube, respectively; HBsAg T-C equals the HBsAg titer of the test tube minus the HBsAg titer of the control tube.

### Variations of HBsAg in the HBsAg-/anti-HBs+/HBeAg+ samples

Under the selective pressure of anti-HBs, HBV genetic variation may occur and produce mutated HBsAg. Thus, the HBV genome region encoding HBsAg was amplified, cloned, and sequenced. In genotype B, D44N, N98T in Pre-S region were found, which was proved to decreased the HBsAg production (Sun et al., 2022). In Genotype C, G73S, I161T, and Del56-116 was found, which were all reported to reduce the extracelluar HBsAg levels (Sengupta et al., 2007; Pollicino et al., 2012; Zhang X. et al., 2018; Sun et al., 2022). As for S region, mutations in all samples occurred predominantly in the major hydrophilic region (MHR, aa 99-169) of HBsAg, especially the “a” determinant (aa 124–147), regardless of genotype B or C. Some mutations also occurred in the transmembrane domains TMD3 and the loop between TMD1 and TMD2. Immune escape-associated mutations and variants that impair HBsAg secretion were commonly seen in these samples. We also found some mutations that haven’t been reported, including Y220F in genotype B, L95W, I126S + T131N + M133T, and F170S + L173R + L175S + Q181R + G185E in genotype C. Common aa substitutions found in HBsAg-/anti-HBs+/HBeAg+ samples were summarized in Tables 3, 4.

**TABLE 3:** Summary of common viral mutations in four HBsAg-/anti-HBs+/HBeAg+ samples of genotype B.

Mutations	Position	Number of samples with the mutation	Number of clones with the mutation	Comments
**Pre-S region (32 clones)**				
D44N	Pre-S1	2	7	Detected frequently in OBI, slightly decrease the intracellular HBsAg in transfected Huh-7 cells (Sun et al., 2022)
N98T	Pre-S1	2	4	Significantly reduced the production of HBsAg *in vitro* and *in vivo* (Sun et al., 2022)
**S region (41 clones)**				
Q101R	MHR	1	10	Immune escape-associated variant (Wang J. et al., 2020, p. 122), detected frequently in OBI (Ye et al., 2019)
P127T	MHR, “a” determinant	2	10	Immune escape-associated variant (Wang J. et al., 2020, p. 122), detected in patients with advanced liver disease (Yamani et al., 2015)
F134L	MHR, “a” determinant	1	14	Immune escape-associated variant (Wang J. et al., 2020, p. 122)
G145R/K	MHR, “a” determinant	2	18	Classical immune escape variant, could reduce the antigenicity of HBsAg and significantly decrease extracellular and intracellular HBsAg level (Pollicino et al., 2014; Wang J. et al., 2020, p. 122)
T131N + M133T	MHR, “a” determinant	2	12	Immune escape-associated variant (Yamani et al., 2015; Kang et al., 2018, p. 131), introducing additional *N*-glycosylation sites and lead to an impaired HBsAg secretion (Zhang et al., 2019)
T131N + M133T + G145R	MHR, “a” determinant	2	10	–
V168A	MHR	2	15	Strongly decreased extracellular HBsAg level in the transfection system (Wang H. et al., 2020)
P178Q	TMD3	2	3	Significantly decreased extracellular HBsAg in transfected Huh-7 cells (Jiang et al., 2022)
Y200F	Loop between TMD3 and TMD4	1	8	–

**TABLE 4:** Summary of common viral mutations in six HBsAg-/anti-HBs+/HBeAg+ samples of genotype C.

Mutations	Position	Number of samples with the mutation	Number of clones with the mutation	Comments
**Pre-S region (49 clones)**				
G73S	Pre-S1	2	15	Detected frequently in OBI, slightly reduced the extracellular HBsAg level *in vitro* (Sun et al., 2022)
Del 56-116^a^	Pre-S1	1	4	Deletion of the pre-S2/S promoter, could lead to an imbalanced HBsAg synthesis and significantly impaired the secretion of HBsAg and viral particles (Sengupta et al., 2007; Pollicino et al., 2012)
I161T	Pre-S2	3	23	Could significantly decrease the serum HBsAg titer in HBeAg-positive pregnant women (Zhang X. et al., 2018)
**S region (80 clones)**				
T47V/R/K	Loop between TMD1 and TMD2	4	34	Detected frequently in OBI (Ye et al., 2019)
P62L	Loop between TMD1 and TMD2	3	22	Identified in patients with chronic HBV infection (Wang et al., 2021)
L95W	TMD2	1	8	–
Q101K/R	MHR	3	30	Immune escape-associated mutation (Wang J. et al., 2020, p. 122), detected frequently in OBI (Ye et al., 2019)
T113N/A	MHR	2	16	Detected in patients with advanced liver disease (Yamani et al., 2015)
I126T/S	MHR, “a” determinant	5	44	Significantly impaired virion and/or HBsAg secretion in both HuH7 cells and mice (Huang et al., 2012) Change tertiary structure of HBsAg and may alter the immunogenicity of HBsAg (Wang et al., 2021)
T131N + M133T	MHR, “a” determinant	2	17	Immune escape-associated variant (Yamani et al., 2015; Kang et al., 2018, p. 13), introducing additional *N*-glycosylation sites, and lead to impaired HBsAg secretion (Zhang et al., 2019)
I126S + T131N + M133T	MHR, “a” determinant	2	16	–
R160S/K	MHR, “a” determinant	3	22	Detected frequently in OBI (Ye et al., 2019)
F170S + L173R + L175S + Q181R + G185E	TMD3	2	12	–

^a^Deletion mutation.

## Discussion

Lumipulse HBsAg-HQ assay is a highly sensitive chemiluminescent enzyme immunoassay (CLEIA) for HBsAg detection. It could dissociate HBsAg from HBsAg-anti-HBs complexes and target both the external structural regions and the internal epitopes of HBsAg, leading to the sensitivity of this assay (≥0.005 IU/ml) being 10-fold higher than the Abbott ARCHITECT assay. This assay is expected to be used for the detecting of OBI and monitoring of HBV activation (Inoue and Tanaka, 2016; Tanaka, 2016). Comparative study and clinical research showed that this assay is sensitive and precise for HBV monitoring during antiviral treatment, and are valuable in predicting the liver tissue pathological states of chronic hepatitis B (CHB) patients (Shinkai et al., 2013; Deguchi et al., 2018; Zhang Z. et al., 2018). In this study, HBsAg in 27.16% (404/1,487) of HBV DNA-positive and HBsAg-negative samples were positive for the HBsAg-HQ assay. Furthermore, 98.8% (170/172) of the positive results were confirmed by the HBsAg neutralization assay, which suggested that the Lumipulse HBsAg-HQ assay has good sensitivity and specificity for detecting OBI samples. As seen in Table 2, the HBsAg dissociation results were completely consistent with the results of the HBsAg-HQ assay, which further indicated that this assay is useful for detecting OBI samples and samples with HBsAg-CICs.

Hepatitis B virus infection is a dynamic process reflecting the interaction between HBV replication and the host immune response. In general, HBV DNA levels in HBeAg-positive individuals are typically greater than 2 × 10^4^ IU/ml (European Association for the Study of the Liver [EASL], 2017; Terrault et al., 2018; Chinese Society of Infectious Diseases et al., 2019). However, the HBV DNA levels of HBsAg-/anti-HBs+/HBeAg+ samples identified in this study were all lower than 2 × 10^4^ IU/ml, suggesting that viral replication was relatively weak compared to the typical HBeAg-positive individuals. In this case, the positive result of anti-HBs indicates that the host has generated strong humoral immune responses to the virus, which reacts with the free HBsAg in plasma and forms CICs, resulting in undetectable HBsAg. Several methods for detecting CICs have been reported (Stanojevic et al., 2000; Zhang et al., 2011; Matsumoto et al., 2017; Wang et al., 2019). Through preliminary experiments, we found that PEG-double precipitation combining HCl and trypsin digestion was better to detect HBsAg-CICs. However, CICs were not found with these methods in three samples (No. 3, 5, and 8). There might be a minute amount of CICs exist in these samples, which were not detected by the current method. A long-term follow-up of these individuals should be further performed to observe the dynamic changes in serological status, as well as HBsAg-CICs.

There are some limitations of this study in whole genome analysis and functional research. Whole genome sequencing of HBV can identify all mutations in the HBV sequence and provide additional clues to explore the relationship between viral genomic variants and the negative HBsAg. However, it requires high quality and concentration of samples. As seen in Table 2, the HBV viral load was between <20 and 300 IU/ml in 9 of 10 samples. We tried but the low concentration and limited sample volume made it fail to perform whole genome sequencing. Compared to the RT, C, and X region of the HBV genome, mutations in the HBsAg coding region were more related to the production of HBsAg and the formation of HBsAg-CICs, so we sequenced the pre-S and S region of HBV. There were many aa substitutions occurred on HBsAg in HBsAg-/anti-HBs+/HBeAg+ samples. Since these samples were all derived from blood donors without antiviral treatment, we speculated that under the dual selection pressure of humoral and cellular immunity, HBsAg has generated immune-escape mutations, such as Q101R, P127T, F134L, G145R, T131N + M133T in genotype B and Q101K/R, I126T/S, T131N + M133T in genotype C. There are many epitopes on HBsAg. Although some epitopes on the “a” determinant mutant, the polyclonal anti-HBs produced in the human body can still recognize and combine to the other epitopes, and form immune complex. Moreover, mutations that lead to the impaired HBsAg secretion, including I126T/S (genotype C), G145R (genotype B/C), T131N + M133T (genotype B/C), and V168A (genotype B) were also identified and may contribute to the negative HBsAg in these samples. Since most of the mutations found in this manuscript were in the MHR region, a region of great interest. Functional researches on varies of mutations have been performed. These studies were summarized and cited in Tables 3, 4. In addition, there are one mutation Y200F and three joint mutations T131N + M133T + G145R, I126S + T131N + M133T, and F170S + L173R + L175S + Q181R + G185E that haven’t been reported before. Further functional research of these mutations will be performed in the future to explore the relationship between them and HBsAg production and secretion.

**FIGURE 3:**
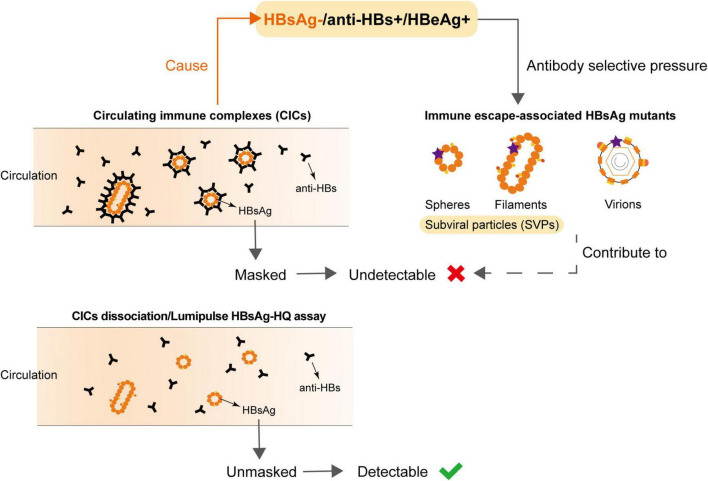
Undetectable HBsAg in HBsAg-/anti-HBs+/HBeAg+ samples was associated with the circulating immune complexes (CICs) and HBsAg variants.

Notably, many studies found that HBV DNA in OBI individuals is only intermittently detected in serum/plasma, and the concentration is low, usually less than 200 IU/ml (Chemin et al., 2009; Spreafico et al., 2015; Mortensen et al., 2016; Candotti et al., 2019). The common mechanisms of HBsAg negativity in OBI include HBV genome variability leading to the changes in HBsAg antigenicity, viral mutations affecting the expression/secretion of HBsAg, host immune control, and epigenetic mechanisms (Pollicino et al., 2007; Pollicino and Raimondo, 2014; Zhu et al., 2016; Raimondo et al., 2019; Sun et al., 2020). In this study, we found the undetectable HBsAg in HBeAg-positive OBI blood donors was mainly because of the formation of HBsAg-CICs, which can be determined by the dissociation methods or Lumipulse HBsAg-HQ assay (Figure 3). Zhang et al. found HBsAg-CICs in two HBV carriers in the clinic who were seroconverted from HBsAg positive and anti-HBs negative to HBsAg negative and anti-HBs positive (Zhang et al., 2011). To our knowledge, this study is the first report providing clear evidence for HBeAg-positive OBI in blood donors, a population that has never received antiviral treatment. Since the hidden relatively active replication of the virus in these individuals is more prone to disease progression and poor prognosis, more clinical attention should be paid to this kind of blood donors.

In conclusion, we identified a minority of OBI blood donors with the HBsAg-/anti-HBs+/HBeAg+ serological pattern. Through using the Lumipulse HBsAg-HQ assay and dissociating HBsAg from CICs, we found the undetectable HBsAg was mainly due to the formation of HBsAg-CICs. HBsAg variants under the host selective pressure may further contribute to the negative HBsAg. These results provide a deeper understanding of the complexity of virus-host interaction during HBV infection, and individuals with the serological pattern need be paid more attention to reduce the risk of disease progression.

## Data availability statement

The original contributions presented in this study are included in the article/Supplementary material, further inquiries can be directed to the corresponding author.

## Ethics statement

The studies involving human participants were reviewed and approved by Bioethics Committees of Beijing Hospital. The patients/participants provided their written informed consent to participate in this study.

## Author contributions

YY, HS, LC, HJ, and LW conceived the study. YY and HS drafted the first version of the manuscript and did data analyses. YY, HS, LC, and LW revised the manuscript. XJ, SS, YX, KF, and AN participated in the study design and helped to draft the manuscript. ZL participated in the collection of samples. All authors contributed to the interpretation of data and approved the final manuscript.
