# Supplementary material for: Circulating immune complexes and mutations of HBsAg are associated with the undetectable HBsAg in anti-HBs and HBeAg positive occult hepatitis B virus infection
Source: Front Microbiol. 2022 Nov 29;13:1063616. doi: 10.3389/fmicb.2022.1063616 (PMC9745333; doi:10.3389/fmicb.2022.1063616)
Supplement: Supplementary file 1 [file Data_Sheet_1.docx]

Table S1. List of primers used in the study

| Primer name | Sequence (5’-3’) | Application |
| --- | --- | --- |
| HBV-2804F | GCCTCATTTTGYGGGTCACCAT | Amplify pre-S region, first round |
| HBV-668R | CTGAGGCCCACTCCCATAG |  |
| HBV-2833F | GGGAACAAGAGCTACAGCATGG | Amplify pre-S region, second round |
| HBV-309R | GGTTGGGGACTGCGAATTT |  |
| HBV-56F | CCTGCTGGTGGCTCCAGTTC | Amplify S region, first round |
| HBrt 1253R | GCAGTATGGATCGGCAGAGGAG |  |
| HBV-178F | CCTAGGACCCCTGCTCGTGTTACAGGC | Amplify S region, second round |
| HBrt 1186R | CCAGTGGGGGTTGCRTCAGC |  |
| M13F | TGTAAAACGACGGCCAGT | Sequencing |
| M13R | CAGGAAACAGCTATGACC |  |

Table S2. Characteristics of the 26 HBsAg-/anti-HBs+/HBeAg- samples

|  |  | HBsAg dissociation^a^ | | | Lumipulse | Abbott ARCHITECT HBV reagent | | | | |
| --- | --- | --- | --- | --- | --- | --- | --- | --- | --- | --- |
| Sample code | HBV DNA  (IU/mL) | HBsAg-T (IU/mL) | HBsAg-C (IU/mL) | HBsAg T-C (IU/mL) | HBsAg-HQ (IU/mL) | HBsAg (S/CO） | Anti-HBs (mIU/mL) | HBeAg (S/CO) | Anti-HBe (S/CO) | Anti-HBc (S/CO) |
| 1 | 23.9 | 0 | 0 | N (0) | N (0.001) | N (0.34) | R (274.56) | N (0.308) | N (1.54) | R (4.4) |
| 2 | ＜20 | 0 | 0 | N (0) | N (0.001) | N (0.21) | R (170.92) | N (0.291) | N (1.83) | R (6.13) |
| 3 | ＜20 | 0 | 0 | N (0) | N (0.001) | N (0.16) | R (486.97) | N (0.261) | N (2.33) | N (0.12) |
| 4 | ＜20 | 0 | 0 | N (0) | N (0.001) | N (0.13) | R (178.69) | N (0.285) | R (0.25) | R (9.9) |
| 5 | ＜20 | 0 | 0 | N (0) | N (0.001) | N (0.4) | R (119.14) | N (0.94) | R (0.07) | R (7.21) |
| 6 | ＜20 | 0.04 | 0.03 | N (0.01) | R (0.067) | N (0.24) | R (182.47) | N (0.288) | R (0.01) | R (9.66) |
| 7 | ＜20 | 0 | 0 | N (0) | N (0.001) | N (0.13) | R (293.4) | N (0.255) | N (1.59) | R (9.58) |
| 8 | ＜20 | 0 | 0 | N (0) | N (0.001) | N (0.12) | R (133.69) | N (0.255) | N (1.6) | R (9.18) |
| 9 | ＜20 | 0 | 0 | N (0) | N (0.001) | N (0.2) | R (256.54) | N (0.392) | R (0.01) | R (7.4) |
| 10 | ＜20 | 0.01 | 0 | N (0.01) | N (0.001) | N (0.22) | R (411.66) | N (0.376) | N (1.85) | N (0.37) |
| 11 | ＜20 | 0 | 0 | N (0) | N (0.001) | N (0.22) | R (364.6) | N (0.218) | R (0.12) | R (10.44) |
| 12 | ＜20 | 0 | 0 | N (0) | N (0.001) | N (0.18) | R (165.08) | N (0.296) | R (0.01) | R (9.72) |
| 13 | ＜20 | 0 | 0 | N (0) | N (0.001) | N (0.17) | R (580.67) | N (0.287) | R (0.02) | R (9.07) |
| 14 | ＜20 | 0 | 0 | N (0) | N (0.003) | N (0.29) | R (135.31) | N (0.419) | R (0.02) | R (8.33) |
| 15 | ＜20 | 0 | 0 | N (0) | N (0.001) | N (0.18) | R (232.23) | N (0.406) | R (0.54) | R (7) |
| 16 | ＜20 | 0 | 0 | N (0) | N (0.001) | N (0.28) | R (260.36) | N (0.323) | N (1.49) | R (9.61) |
| 17 | ＜20 | 0 | 0 | N (0) | N (0.001) | N (0.367) | R (256.2) | N (0.078) | N (1.49) | N (1.13) |
| 18 | ＜20 | 0 | 0 | N (0) | N (0.001) | N (0.18) | R (414.31) | N (0.259) | R (0.01) | R (9.27) |
| 19 | ＜20 | 0 | 0 | N (0) | N (0.003) | N (0.11) | R (363.61) | N (0.232) | R (0.01) | R (8.44) |
| 20 | ＜20 | 0 | 0 | N (0) | N (0.001) | N (0.14) | R (130.9) | N (0.283) | N (1.56) | N (0.8) |
| 21 | ＜20 | 0 | 0 | N (0) | N (0.001) | N (0.32) | R (223.68) | N (0.462) | R (0.26) | R (8.39) |
| 22 | ＜20 | 0 | 0 | N (0) | N (0.001) | N (0.23) | R (228.13) | N (0.318) | N (1.46) | R (5.97) |
| 23 | ＜20 | 0 | 0 | N (0) | N (0.001) | N (0.12) | R (>1000) | N (0.295) | N (1.73) | N (0.1) |
| 24 | ＜20 | 0 | 0 | N (0) | N (0.003) | N (0.13) | R (156.26) | N (0.298) | R (0.01) | R (3.04) |
| 25 | ＜20 | 0 | 0 | N (0) | N (0.001) | N (0.17) | R (>1000) | N (0.439) | N (1.1) | R (6.74) |
| 26 | ＜20 | 0 | 0 | N (0) | N (0.001) | N (0.12) | R (200.65) | N (0.296) | N (1.79) | N (0.05) |

a: HBsAg-T and HBsAg-C represent HBsAg titer of test tube and control tube, respectively; HBsAg T-C equals the HBsAg titer of the test tube minus the HBsAg titer of the control tube.
